# Analytical Model for the Fatigue Analysis of Steel Joints by Clamps According to the Lever Length

**DOI:** 10.3390/ma14247726

**Published:** 2021-12-14

**Authors:** Manuel Cabaleiro, Rafael Comesaña, Cristina González-Gaya, Carlos Caamaño

**Affiliations:** 1 Department of Construction and Manufacturing Engineering, Universidad Nacional de Educación a Distancia (UNED), C/Juan del Rosal 12, 28040 Madrid, Spain; cggaya@ind.uned.es; 2 Department of Materials Engineering, Applied Mechanics and Construction, School of Industrial Engineering, University of Vigo, 36208 Vigo, Spain; racomesana@uvigo.es (R.C.); jccaam@uvigo.es (C.C.)

**Keywords:** clamp, new joint, reconfigurable joint, bolt, fatigue, dynamic load, deconstruction

## Abstract

Among the most commonly used materials in the construction of structures in the last two centuries are iron and steel. Clamp joints are a suitable type of joint when it is necessary to rehabilitate or modify a historical steel structure for new uses, reinforcing or modifying it with new beams, without the need to drill or weld on the original structure. The clamps allow beams to be joined with a flange (such as I-beams) without the need for any prior operation on the beams and allow the manufacture of completely removable and reconfigurable structures. Developing and analysing this type of fully removable and reconfigurable structure is necessary. To date, no studies have been carried out on the fatigue behaviour of steel joints by clamps, especially taking into account their main geometric characteristics, such as the size of the clamp levers. In this work, an analytical model is proposed that allows for the analysis of the number of cycles and the fatigue limit of clamp joints as a function of the size of the clamp levers. In addition, various fatigue tests are performed with different clamp sizes. The experimental results are compared with those obtained with the proposed methodology. Finally, the relationships between the lever length and the fatigue behaviour of the clamp joints have been determined. It is concluded that an increase in the size of the front lever is associated to a decrease in the fatigue limit. On the contrary, if the size of the rear lever is increased, the fatigue limit of the joint increases. In general, according to the obtained results, the resistance of the joint can be reduced to approximately one third when it is subjected to fatigue loads.

## 1. Introduction

There are a large number of steel structures around the world that are still in service or whose rehabilitation for new uses is planned. These structures include bridges, pavilions, metro stations, and train stations. When the rehabilitation, modification, or strengthening of a steel structure is required due to a new purpose, it is often necessary to reinforce or reconfigure it with new beams. As these structures constitute historical heritage, invasive work, such as drilling or welding, cannot be carried out. In addition, structural joint fatigue damage is one of the main problems experienced by these structures, especially those exposed to dynamic loads, such as bridges. Regarding fatigue damage in historic steel bridges, there are several works, such as the one reported by El-Sisi et al. [1], where field measurements were addressed to determine the real dimensions of an aged, riveted steel bridge. From these measurements, finite element models were created to calculate the fatigue life of the bridge under various loading conditions. The work of Marques et al. [2] describes the experimental and numerical studies carried out by the Vibrations and Monitoring Laboratory (ViBest) of FEUP. Evaluation of the dynamic effects and fatigue analysis of an old Portuguese riveted railway bridge, the Trezói Bridge, was addressed. For this purpose, complementary numerical and experimental approaches were applied to assess the fatigue effects. Finally, the obtained results were used to calculate the residual fatigue life for this bridge. Moreover, in the work of Lehner et al. [3], a method is proposed for the analysis of fatigue damage of the support truss of a riveted steel overhead crane.

**Figure 1:**
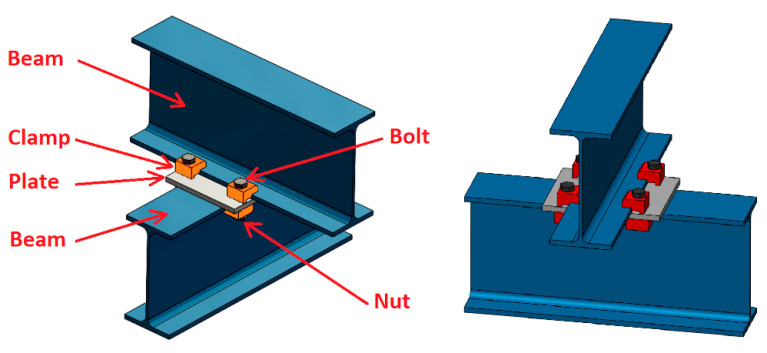
Example of clamped joint with its component elements.

The maintenance and the reinforcement of these historical structures is a fundamental requirement to increase their useful life, aiming to reduce the environmental impact due to the construction of new structures and preserving the historical heritage. In those historical steel structures that need to be reinforced or modified with new profiles, invasive operations are not allowed, so the use of clamp joints constitutes a promising alternative (Figure 1).

Clamp joints are fundamentally composed of a clamp, a bolt, and a nut, which are employed to join two beams or a plate (previously drilled) to a beam (Figure 1). With this type of joint, any type of previous operation on a profile with flanges is not necessary. There are several clamp types and manufacturers [4,5,6]. Clamp dimensions may differ between different models, but the lengths of the front lever and the rear lever mainly determine the clamp operation.

**Figure 2:**
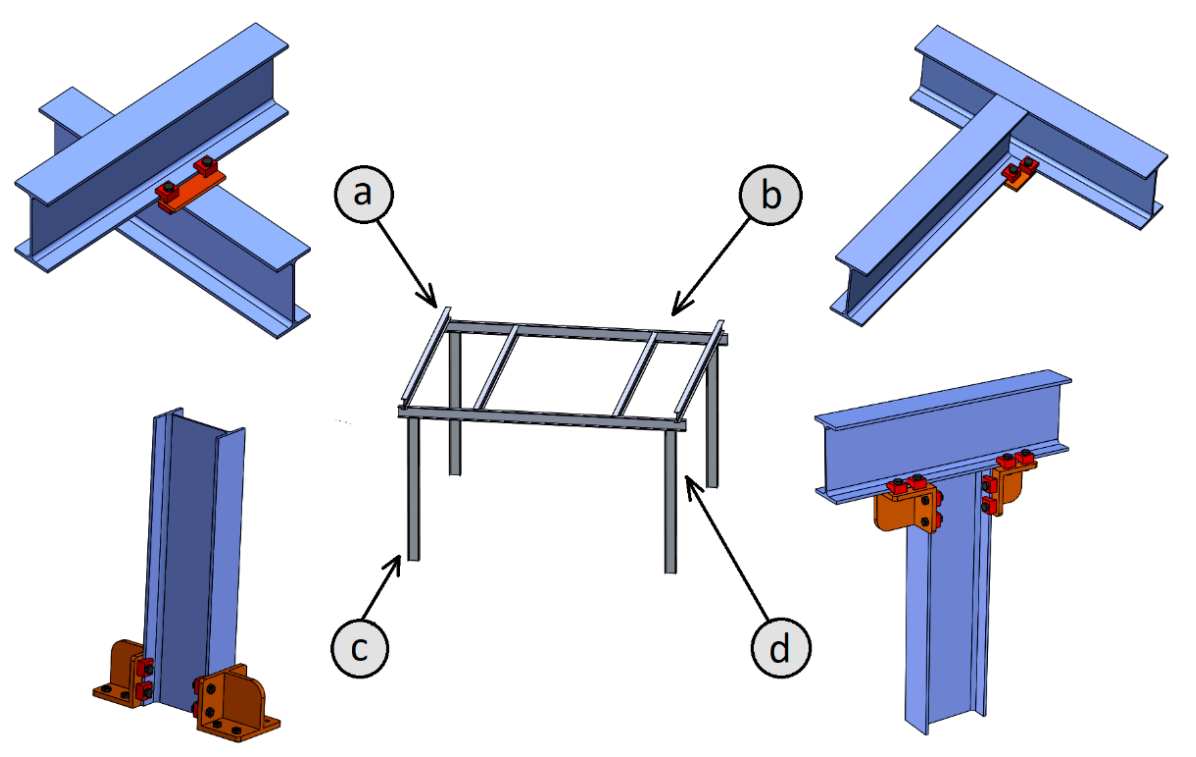
Some examples of joints with clamps: (**a**) cross union between beams; (**b**) union between beam end and beam web; (**c**) removable base system for I-section pillars; (**d**) union of end beam and pillar at 90º.

In addition to allowing implementations in historical steel structures, this type of clamp joint also enables the construction of fully dismountable and reconfigurable structures. Currently, welded joints are the most common joining systems in steel structures, but these joints are not removable. Classic bolted joints are also widely used. This kind of joint is removable, but not very easily reconfigurable since each beam always needs some prior invasive work, such as drilling, stiffening, welding of head plates, etc. Thus, each element of the structure is valid for a limited number of new structure configurations. The joints with clamps allow the manufacture of totally dismountable and reconfigurable structures, where it is not necessary to carry out any previous operation on the beams. Thus, both the beams and clamps are completely reused each time the structure needs to be reconfigured. By means of clamp joints, different joint configurations between I-section beams can be made. Figure 2 shows some examples of joints with clamps: (a) cross union between beams; (b) union between beam end and beam web; (c) removable base system for I-section pillars; (d) union of end beam and pillar at 90°.

The development and analysis of this type of structure is necessary to allow progress towards more sustainable and environmentally friendly structures. Thus, it is not necessary to scrap the structures each time they are adapted to a new purpose. In addition, the reconfigurable structures allow significant economic savings. Some research works such as Basta et al. [7] have already focused on design for deconstruction (DfD) as one of the most effective end-of-life structure design scenarios, aiming at the design of reversible buildings. Other works, such as that of Eckelman et al. [8], have analysed the energy and environmental benefits of design for deconstruction (DfD) with the aim of reusing durable construction components in steel buildings.

There are few research studies about clamp joints, among them the work of Cabaleiro et al. [3], who carried out the first study of the behaviour of clamped joints based on the dimensions of the clamps. For this purpose, an analytical model based on the T-stub model of Eurocode 3 is proposed. Later, Cabaleiro et al. [9] proposed an analytical model for the calculation of clamp joints based on the preload applied to the bolts. This type of joint with clamps can be used in the modification of structures subjected to dynamic loads, such as historical railway bridges or structures for industrial facilities (e.g., on the automotive plants facilities and structures which must frequently support dynamic loads due to machinery and equipment [9]). Therefore, it is necessary to assess the fatigue behaviour of this type of joint, especially depending on the features that determine its operation (the lever lengths). There are numerous research papers on the behaviour of classic bolted connections [10,11,12,13,14,15,16,17], many of them focused on fatigue. For example, Zampieri et al. [18] carried out a review of the fatigue resistance of bolted shear connections, Bartsch et al. [19] performed an analysis of the fatigue strength of end plate connections with prestressed bolts, and Liu et al. [20] showed a simplified modelling strategy based on continuous damage mechanics for the evaluation of cumulative fatigue damage in metal bolted joints. 

Moreover, in the work of Yan et al. [21], a test and finite element analysis of a new type of double-limb double-plate connection joint in narrow base tower is shown. However, to the best of the authors’ knowledge, at present, no studies have yet been carried out on the fatigue behaviour of clamp joints, especially taking into account their main geometric characteristics, such as the size of the clamp levers. On the other hand, it is also necessary to develop fatigue calculation methods for this type of joint that are easily applicable by engineers for the calculation and selection of the clamp levers size to be used.

Hence, the objective of this work is to propose an analytical model that allows a quick analysis of the number of cycles and the fatigue limit of clamped joints and their bolts, as a function of the size of the clamping levers. This analytical model will make it possible to analyse the fatigue behaviour of this type of joint. Experimental tests are carried out for the validation of the proposed methodology.

## 2. Proposed Analytical Method

In this work, an analytical model is proposed for the calculation and fatigue analysis of clamp joints and their bolts subjected to alternating stresses. The model is applied to high cycle fatigue (greater than 1000 cycles [22]) and takes into account the diameter and grade of the bolt and the geometric properties of the clamp lever. These are the most commonly varied parameters in clamp joints. In all cases, it is considered that the joint presents an unlimited life when 10^6^ cycles are reached without failure [22]. The clamp is assumed to be rigid and not considered in the fatigue calculation because the analysis is focused on the bolt fatigue limit.

The steps followed for the development of the analytical model for fatigue calculation of clamped joint bolts are presented below:

### 2.1. Calculation of the Theoretical Value of the Bolt Fatigue Limit

The calculation of the theoretical value of the bolt fatigue limit (without considering the fatigue notch factor) is according to the following equation

*S_e_* = *C_a_* × *C_b_* × *C_c_* × *C_d_* × *C_e_* × *S’_e_* (1)

where

*S_e_* = theoretical bolt fatigue limit (not considering the notch factor)

*S’_e_* = theoretical fatigue limit of the steel used (for the usual steels of bolts in this type of joint, this is 0.5 *S_ut_* [23], where *S_ut_* is the ultimate tensile strength of the material)

*C_a_* = coefficient for surface finish (for the commonly used cold-rolled bolts this is 0.750 [1])

*C_b_* = coefficient by size, which, depending on the diameter *d* (mm) of the bolt, is according to the equation [22]

(2) $$C_{b}=0.869d^{-0.097}$$

*C_c_* = confidence coefficient (which, for a 99% reliability, is 0.814 [22])

*C_d_* = temperature coefficient, which for working temperatures below 450 °C is 1.00

*C_e_* = load coefficient, which in clamp joints, due to the bolts having a bending stress component, is 1.0 [22].

### 2.2. Calculation of the Load Experienced by the Bolt

**Figure 3:**
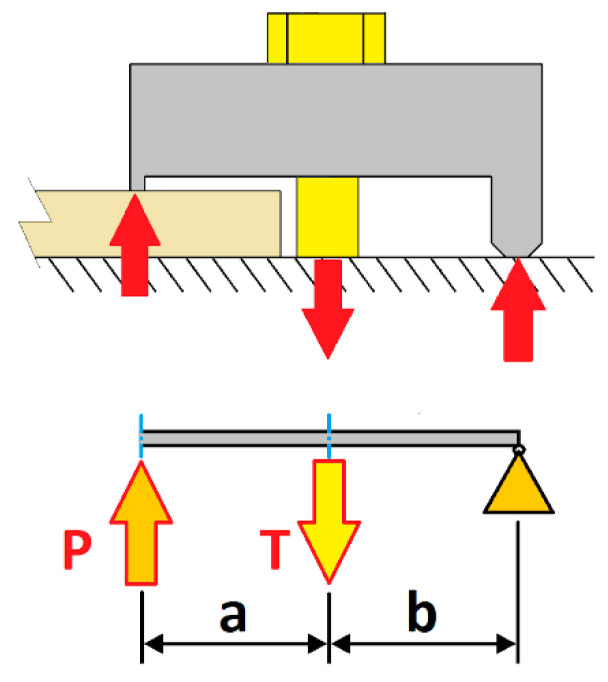
Diagram of the clamp operation (lever effect).

The force T experienced by the bolt is directly related to the lever effect (Figure 3). This value *T* depends on the load *P* [3] applied on the clamp edge and the geometric parameters of the clamp levers (parameters *a* and *b*). For the case of clamp joints, the value of *T* can be defined according to Equation (3):

(3) $$T=P\frac{a+b}{b}$$

Similar to the behaviour of classic bolted joints, the preloaded bolt bears only a small part of the load *T* applied to a preloaded joint, and this load portion is a function of the joint stiffness constant *C* [22]. Taking into account the lever effect, the expression that determines the load *Pb* experienced by the bolt as a function of the total load *P* applied to the clamp is:

(4) $$Pb=C\cdot T=C\left(P\frac{a+b}{b}\right)$$

### 2.3. Calculation of the Maximum Allowable Alternating Stress in the Bolt

**Figure 4:**
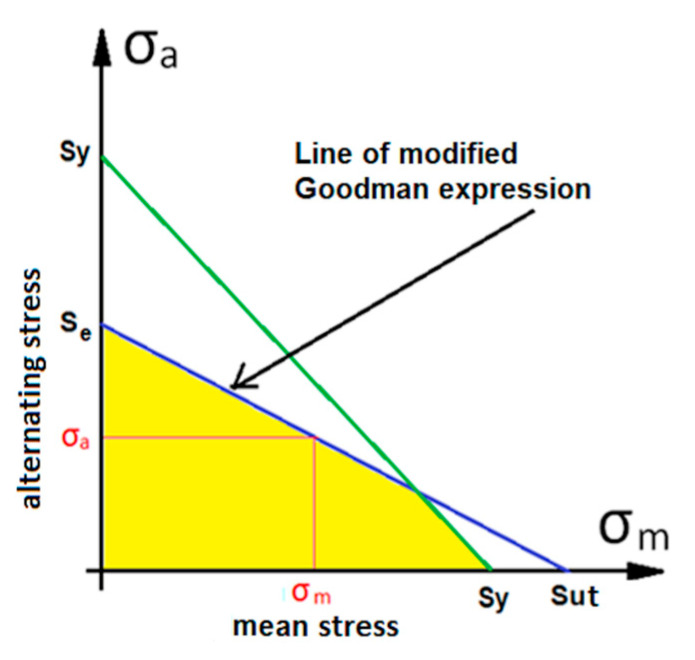
Graph of the relationship between mean stress *σ_m_* supported by the bolt and maximum allowable alternating stress *σ_a_*. In this graph *S_ut_* is the ultimate tensile strength and *S_e_* is the stress for unlimited life of the material (10^6^ cycles in this case) and *S_y_* the yield strength of the material.

For the calculation of the maximum admissible alternating stress, the modified Goodman expression [22] is applied. This expression (Equation (5)) allows for the calculation of the maximum alternating stress while remaining on the safe side:

(5) $$\sigma_{a}=S_{e}\left(1-\frac{\sigma_{m}}{S_{ut}}\right)$$

where *σ_a_* is the alternating stress, *σ_m_* the mean stress supported by the bolt, *S_ut_* the ultimate tensile strength of the material, and *S*_e_ the theoretical fatigue limit of the bolt. Furthermore, Equation (5) is combined with the yield strength (*S_y_*) line of the bolt material (see Figure 4).

For this type of joint and according to the lever effect of the clamp, for a preload stress of the bolt *σ_prec_*, a resistant area (stress area) of the bolt *A_t_*, a factor *K_f_* (fatigue concentration factor for the thread or fatigue notch factor [23]), and a coefficient *C* of joint stiffness, the equations to calculate the alternating stress (*σ_a_*) and the mean stress (*σ_m_*) experienced by the bolt are:

(6) $$\sigma_{a}=\frac{Pb\;}{2A_{t}}=\frac{C\;K_{f}}{2A_{t}}\left(P\frac{a+b}{b}\right)$$

(7) $$\sigma_{m}=\sigma_{prec}+\sigma_{a}$$

According to Equation (5), the maximum allowable alternating stress (*σ_ac_*) for each mean stress (*σ_m_*) of the bolt can be calculated for each clamp load *P*. Moreover, using Equations (1), (6), and (7), the alternating stress calculated (*σ_ac_*) is a function of the preload stress (*σ_prec_*), the bolt’s ultimate tensile strength (*S_ut_*), the bolt’s resistant area (*A_t_*) and the clamp lever parameters (*a* and *b*) according to Equation (8) for a clamp load *P*:

(8) $$\sigma_{ac}=S_{e}\cdot \left(1-\frac{\sigma_{m}}{S_{ut}}\right)=0.2976\;S_{ut}\cdot \left(1-\frac{\sigma_{prec}+K_{f}\;\frac{C\;}{2\;A_{t}}\left(P\frac{a+b}{b}\right)}{S_{ut}}\right)$$

Taking into account that the applied load *P* must not exceed the preload stress of the bolt (*P_L_*), if the load *P* is taken as the maximum allowable load that produces a *P_L_* value on the bolt, the maximum allowable alternating stress (*σ_ac_max_*) for an unlimited bolt life is according to Equation (9):

(9) $$\sigma_{ac\_max}=0.2976\;S_{ut}\cdot \left(1-\frac{\sigma_{prec}+K_{f}\;\frac{C\;P_{L}}{2\;A_{t}}\left(\frac{a+b}{b}\right)}{S_{ut}}\right)=0.2976\;S_{ut}\cdot \left(1-\frac{\left(\sigma_{prec}\;\left[1+K_{f}\;\frac{C\;}{2\;}\left(\frac{a+b}{b}\right)\right]\right)}{S_{ut}}\right)$$

### 2.4. Calculation of the Bolt’s Useful Life

To calculate the useful life according to the maximum alternating load experienced by the bolt, the Wöhler expression (Equation (10)) is used:

(10) $$\frac{S_{ut3}-S_{e}}{log\;10^{6}-log10^{3}}=\frac{S_{ut3}-S_{x}}{log\;\left(N_{x}\right)-log10^{3}}$$

For this application, *S_ut3_* is according to Equation (11):

(11) $$S_{ut3}=\frac{0.9S_{ut-}\sigma_{prec}}{2}$$

where *S_ut_* is the ultimate tensile strength of the material for 10^3^ cycles and *σ_pre_* is the preload stress applied to the bolt.

**Figure 5:**
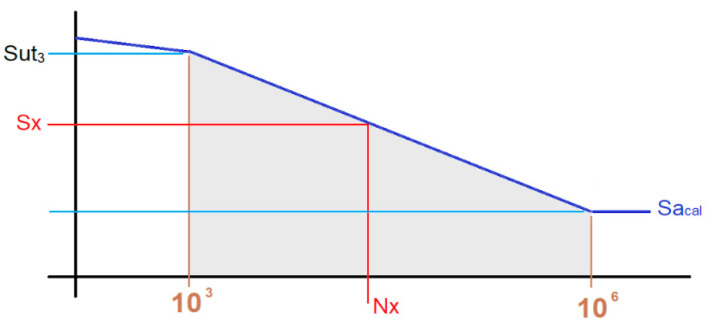
S-N diagram, where *S_ut3_* (material fatigue limit stress value for 10^3^ cycles) and *S_e_* are the calculated maximum alternating stress.

Therefore, according to Equation (10), the value of the additional load *S_x_* (Figure 5) that it supports by the bolt for a number of life cycles *N_x_* is:

(12) $$S_{x}=S_{ut3}-\left[\left(log\;\left(N_{x}\right)-3\right)\left(\frac{S_{ut3}-\sigma_{ac\_max}}{3}\right)\right]$$

where *σ_ac_max_* is the maximum permissible alternation value for unlimited life (Equation (9)).

Based on Equation (10), the expression that gives the number of cycles reached by the joint as a function of the additional load *S_x_* supported by the bolt is:

(13) $$N_{x}=10^{\left(\frac{\sigma_{ac\_max}-S_{x}}{\sigma_{ac\_max}-S_{ut3}}\right)3+3}$$

where, in addition, the load *P* applied to the joint can be obtained from Equation (6):

(14) $$P=\frac{S_{x\cdot }At}{C\;K_{f}}\left(\frac{b}{a+b}\right)$$

such that, for the case of 10^6^ cycles (i.e., unlimited life), the equation that gives the maximum load (*P_max_*) to apply is:

(15) $$P_{max}=\frac{\sigma_{acalmax}\;At}{C}\left(\frac{b}{a+b}\right)$$

## 3. Materials and Experimental Methods

### 3.1. Laboratory Test

**Figure 6:**
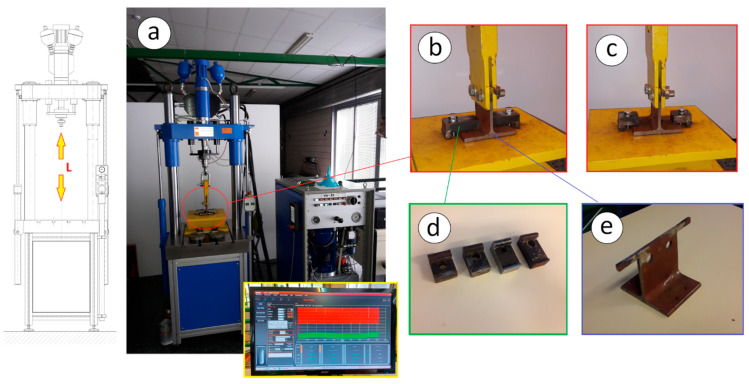
Laboratory tests: (**a**) universal hydraulic tension-compression fatigue testing machine; (**b**) long clamp test; (**c**) short clamp test; (**d**) clamp sizes used; (**e**) specimen of T-shape made from an IPE220.

**Figure 7:**
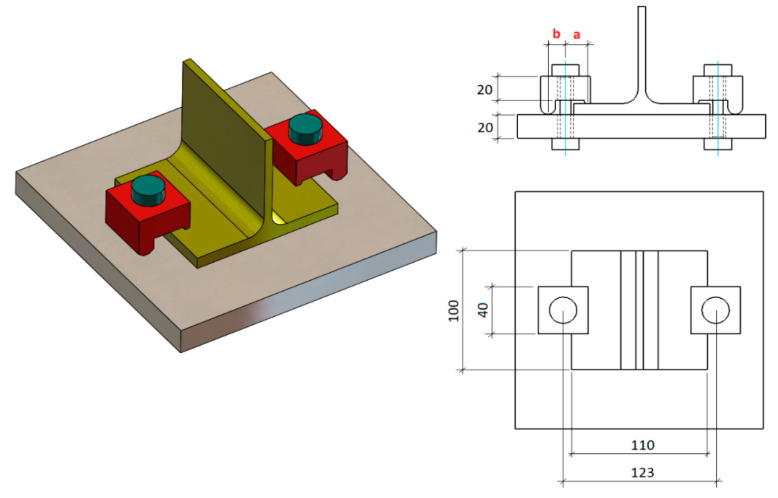
Drawings of the tested joint with general dimensions (mm).

To validate the proposed analytical model, several experimental tests were carried out. For these tests, a universal hydraulic axial fatigue testing machine of the brand “walter + bai ag” of the LFV series was used, with a load capacity of 25 kN and a load frequency of 10 Hz. Several specimens were manufactured in a T-shape made from an IPE220 laminated profile cut along the web (Figure 6 and Figure 7). The specimens were made of S235 steel, with a 100 mm length. The T-shaped specimens were fixed by two clamps to a fixed plate rigidly anchored to the bench of the press table.

To assess the variation of the fatigue limit and achieved number of cycles as a function of the clamp size, four different sizes of clamps were used. The rear lever (b) had a fixed value of 17 mm for all clamps, and four different values were used for the front lever (a): 19, 29, 39, and 44 mm (Figure 6 and Figure 7). The bolts used were ISO 4014 M10 and grade 8.8 (yield strength: 6.40e + 008 MPa; ultimate strength: 8.00e + 008 MPa). The load (*L*) was applied in the centre of the T-shaped specimen, which was fixed by means of the two clamps (Figure 6). The clamps were 20 mm thick and 40 mm wide (S375 steel) to guarantee that they would not fail by fatigue (this point was calculated previously by FEM simulation). Taking as a starting point the previously calculated values of the fatigue limit load by the proposed analytical method, different loads were applied to the joint, and the number of cycles reached before its fatigue failure was experimentally obtained.

The experimental results were used to validate the results calculated by the proposed analytical model. The tightening torque applied to the M10 8.8 bolts was controlled by a torque wrench with a measurement accuracy of 4%, applying the corresponding preload according to Eurocode 3 [24]. In addition, strain gauges with a length of 1.60 mm, a gauge factor of 2.16, and 120.00 Ω resistance were used to verify the preload. For data acquisition, a Vishay D4 strain data acquisition system with a quarter-bridge gauge configuration was employed.

Fracture surface analysis in the failed bolts due to fatigue tests was performed by means of optical microscopy (SMZ1000, Nikon Metrology, Brighton, MI, USA). Surface topography analysis was performed by interferometric profilometry (Filmetrics Profilm3D-200, KLA, San Diego, CA, USA). Although the lateral resolution is limited, this technique makes it possible to observe the fracture surface with high vertical resolution.

### 3.2. Analysis with the Analytical Model

According to the analytical model proposed in this work, analysis of the load cycles and fatigue limit of a clamp joint was performed for different geometric values of the clamp lever and with M10 (grade 8.8) bolt size. The front lever analysed values were a = 19, 29, 39, 44, and 49 mm, while the considered values for the rear levers (b) were 17, 27, 37, and 47 mm.

**Figure 8:**
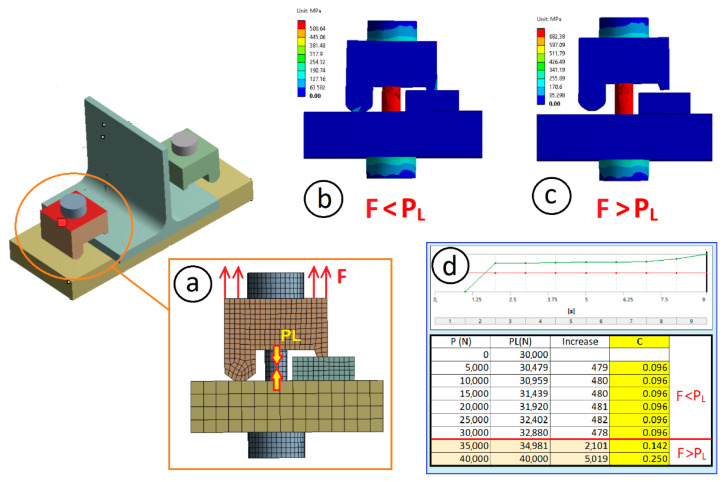
Calculation by simulation of the coefficient *C* of stiffness of the joint: (**a**) applied forces and meshing of the model; (**b**) stresses and deformation when the applied load F is less than the preload of the bolt; (**c**) stresses and deformation when the applied load F is greater than the preload of the bolt; (**d**) results obtained with the simulation and obtaining the (*C*) stiffness coefficient.

As the joint stiffness constant C is an analytically value difficult to find for this type of clamp joint, previous modelling of a joint with clamp and bolt was performed, and the stiffness (and the corresponding C value) was calculated by FEM simulation. For this purpose, a joint bolt model with identical length and *A_t_* area to the real bolt was employed. Moreover, clamps with same lever dimensions a and b were used to avoid potential calculation asymmetries (see Figure 8). Moreover, the thickness of the anchor plate, clamp, and the flange of the T were modelled with the actual thickness used in this work. ANSYS^®^ software was used for the numerical modelling process. First-order hexahedral elements were employed in all parts of the model. The element sizes used were: 5.0 mm for the base and 2.0 mm for the T-shape, clamp, and bolt. A preload of 30 kN was applied to the bolt.

## 4. Results and Discussion

### 4.1. Results of Experimental Tests

**Figure 9:**
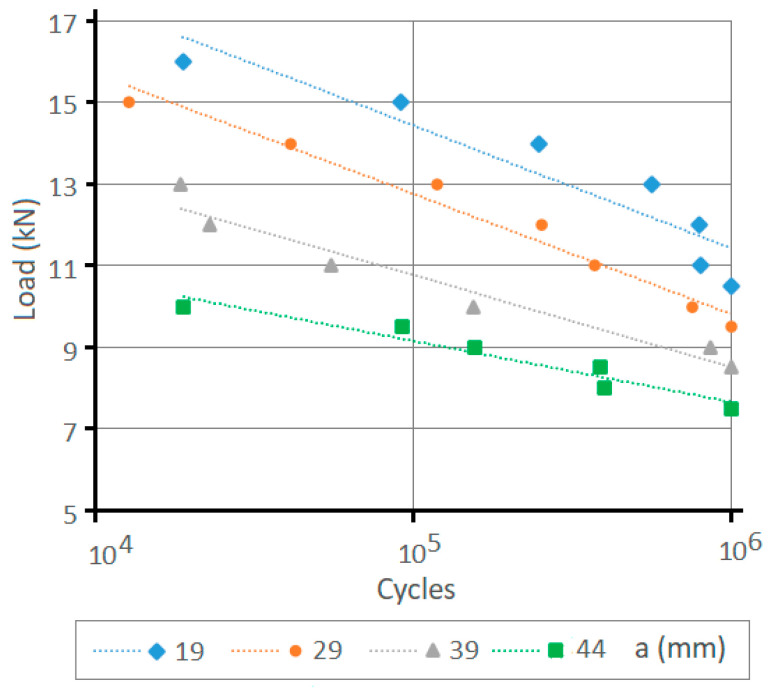
Result of the number of life cycles reached in the laboratory as a function of the front clamp lever (*a*) and as a function of the load (*L*) applied to the joint. The fatigue test is stopped whenever the number of load cycles reaches a value of 10^6^ without the specimen breaking.

The results obtained during the experimental tests are shown in Figure 9. This graph shows how the number of cycles achieved before fatigue failure for a fixed load decreases as the front clamp lever is increased. Moreover, as expected as the load applied to the clamp increases, the number of cycles supported by the joint decreases, being similar for the four lever sizes tested.

**Figure 10:**
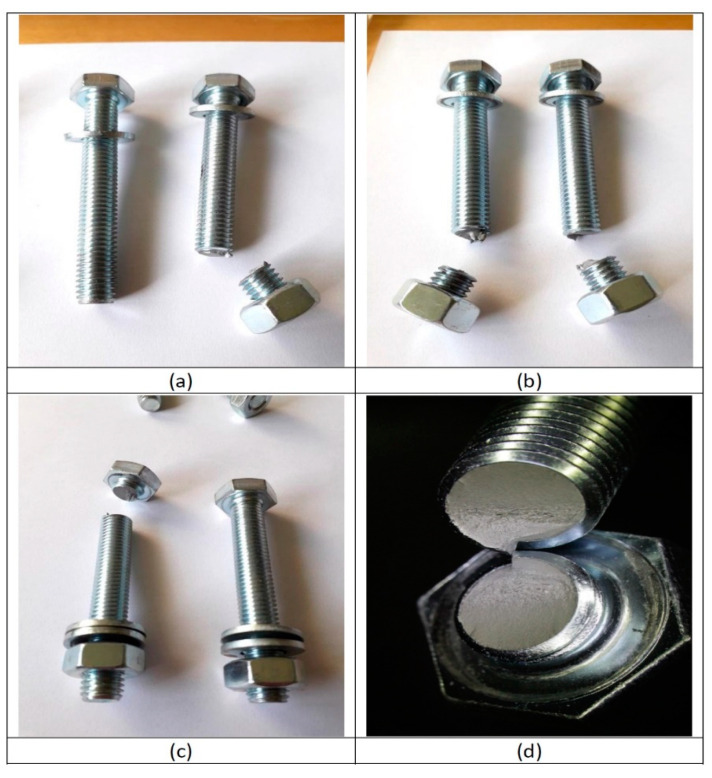
Bolt failure: (**a**) failure of one bolt at nut location; (**b**) failure of both bolts at nut location; (**c**) failure of one bolt at head location; (**d**) appearance of the fracture surface.

The experimental tests carried out in the laboratory revealed that 96% of the fracture surfaces were in the lower part of the bolt, at the bolt–nut union zone (see Figure 10). Failure of one bolt at the head location is observed in the remaining 4% of experimental tests.

**Figure 11:**
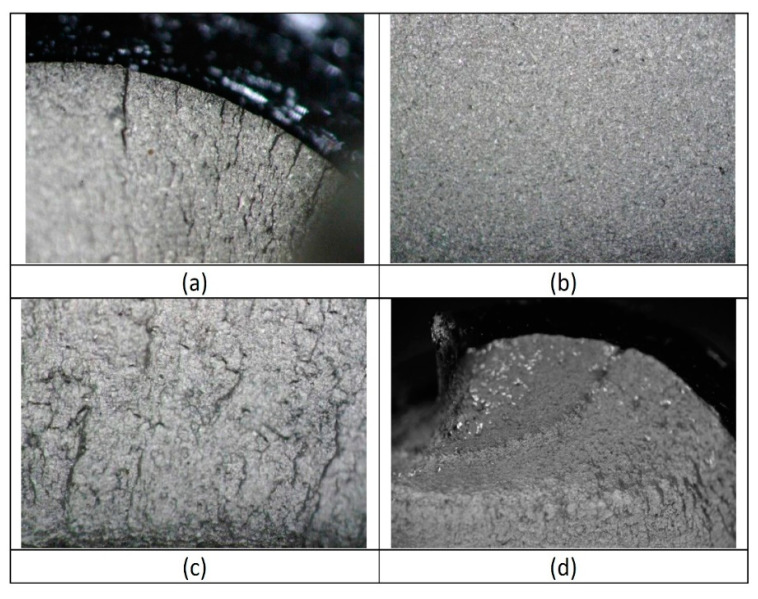
Fracture surface of the bolts: (**a**) multiple initiation sites and ratchet marks due to crack surface convergence; (**b**) relatively smooth surface of stable crack growth; (**c**) rough fracture surface; (**d**) shear lip.

Figure 10 shows the appearance of the fracture to the naked eye, and Figure 11 shows the optical micrographs of the typical fracture surface. The fatigue cracks are produced at the thread root, from the side of the clamped plate. The fracture surface shows several ratchet marks, suggesting multiple crack initiation sites on different planes at the thread root. Multiple cracks converge at a relatively smooth surface corresponding to the stable crack growth region, which extends over most of the fracture surface. The fracture surface continues with a rougher area that could be attributed to a fast intergranular fracture or to an increased crack growth rate and is followed by a noticeable shear lip at approximately 45 degrees to the cross-section. The ductile characteristic behaviour of the bolt material, and the apparent material plastic deformation at higher magnification, suggest that the rough surface corresponds to an increased crack growth rate.

**Figure 12:**
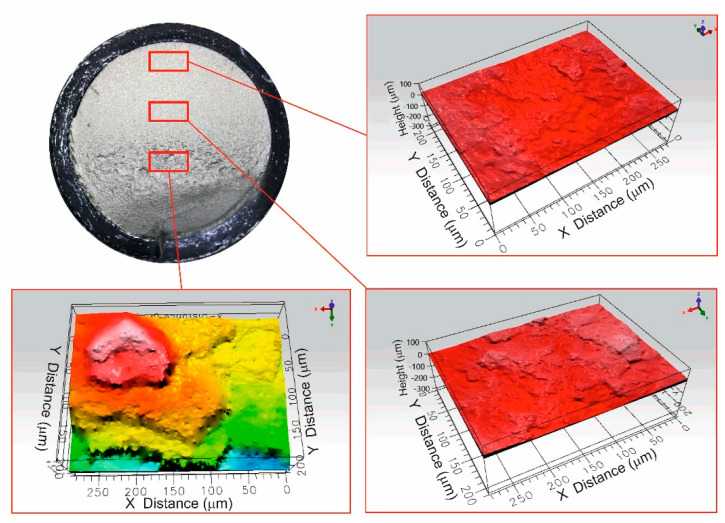
Shows representative fracture surfaces obtained with the different clamping lengths, for high loads, medium loads, and low loads (increased number of cycles to failure). It can be observed how the stable crack growth region increases as the load is decreased. Moreover, the rough surface extension is consistently reduced with the load reduction.

Figure 12 shows the topography of the fracture surface obtained by an interferometric microscope. Although the lateral resolution of this technique is limited, the three-dimensional profiles allows to observe the characteristic topography produced by the plastic deformation during the stable crack growth. The surface roughness increases from *Sa* = 5.46 µm close to the crack initiation boundary to *Sa* = 53.87 µm at the surface adjacent to the shear lip, where a more abrupt topography is observed.

**Figure 13:**
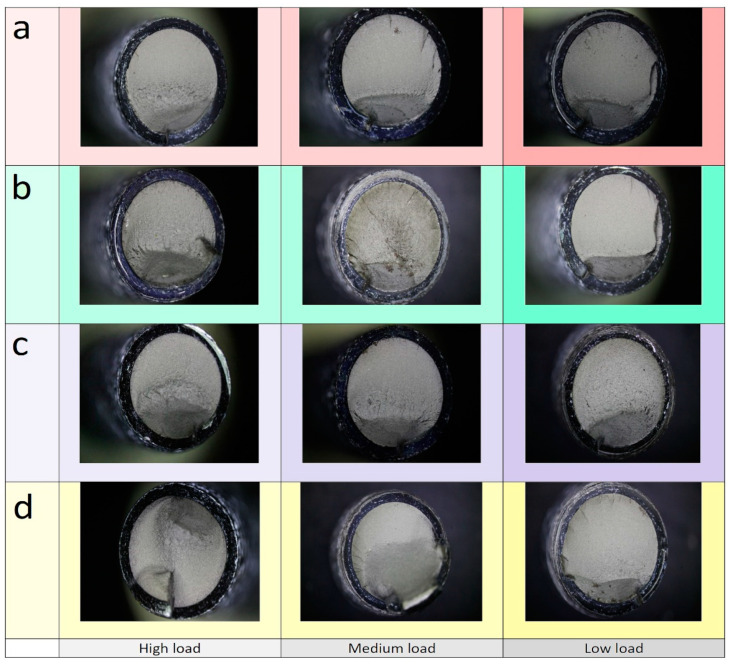
Fracture surface produced by different clamp lengths at high, medium and low loads. (**a**) levers of (*a/b*) 19/17 mm (**b**) levers of (*a/b*) 29/17 mm (**c**) levers of (*a/b*) 39/17 mm (**d**) levers of (*a/b*) 44/17 mm.

Figure 13 shows representative fracture surfaces obtained with the different clamping lengths, for high loads, medium loads, and low loads (increased number of cycles to failure). It can be observed how the stable crack growth region increases as the load is decreased. Moreover, the rough surface extension is consistently reduced with the load reduction.

**Figure 14:**
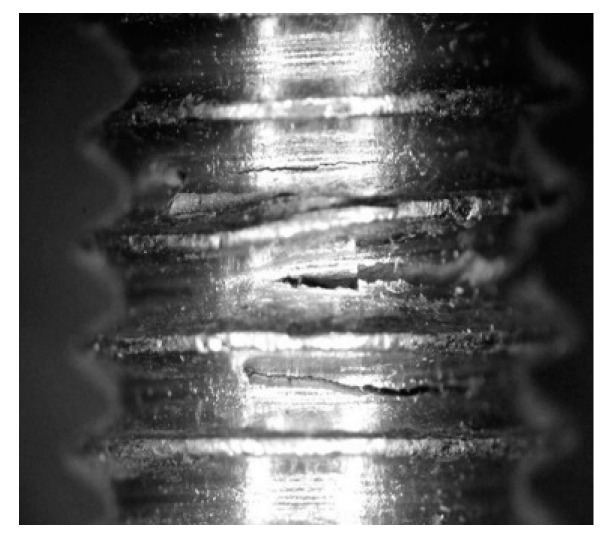
Cracks generated at consecutive thread roots in longer clamp configuration and low load conditions.

The observation of the thread’s lateral surface shows that, as a general rule, the fatigue crack is located in only one thread root. Nevertheless, additional cracks grown from the adjacent roots are observed in failures produced using the longer clamps (see Figure 14). This effect can be attributed to a less homogeneous load transfer at the bolt head when using the joint with longer clamps and led to particular fracture surfaces at low cycle numbers or high loads. In these bolts, as the cracks grow from several roots, some fracture surfaces grow from the clamped plate side to the bolt centre, but other quasi-parallel fracture surfaces are produced by cracks growing from the front and rear sides to the bolt centre. The resulting fast fracture surface is located at the clamped plate side (see Figure 13, 44/17 clamps, high load).

### 4.2. Analytical Model Results

**Figure 15:**
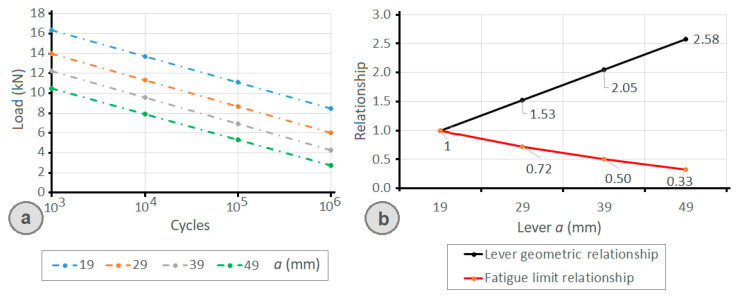
(**a**) Limit load to fatigue by the clamp (*b* = 17 mm) determined by the proposed analytical expression as a function of the front lever (*a*) of the clamp and as a function of the number of life cycles; (**b**) relationship between the increase in the value of the front lever (*a*) compared to the shortest front lever (*a* = 19 mm).

The *S_e_* value for the calculation of the unlimited life value of the material for the bolt grade 8.8 with *S_ut_* = 800 MPa was *S_e_* = 237.4 MPa. The expression indicated by Eurocode 3 [23] for the bolt preloading led to the preloading stress of 509 MPa. The stiffness *C* of the joint was 0.096 according to the results obtained with the FEM simulation (see Figure 8). Using Equation (12) for the values of the front lever (*a*) of 19, 29, 39, and 49 mm and a rear lever (*b*) value of 17 mm, the maximum joint allowable load for a duration of between 10^3^ and 10^6^ cycles is shown in Figure 15a and Table 1. According to the graph in Figure 15a, as the size of the front lever (*a*) increases and for a fixed rear lever (*b*), the fatigue resistance (i.e., the number of cycles that the joint can withstand) decreases.

As can be seen in Figure 15b, an inverse relationship between the length increase of the front lever (with respect to the shortest front lever *a* = 19 mm) and the fatigue limit increase is obtained. In other words, as the value of the front lever (*a*) is increased, the fatigue load limit decreases. It must be highlighted that this relationship is not directly proportional, i.e., an increase to double the length of the front lever does not imply a decrease to half the limit of fatigue resistance, as shown by Figure 15b.

**Figure 16:**
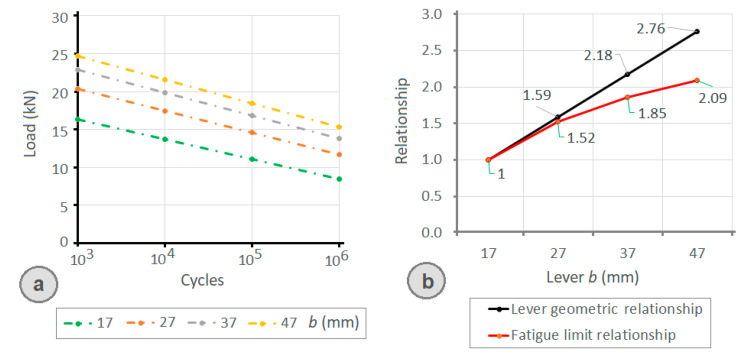
(**a**) Limit load to fatigue by the clamp (*a* = 19 mm) determined by the proposed analytical expression as a function of the rear lever (*b*) of the clamp and as a function of the number of life cycles; (**b**) relationship between the increase in the value of the rear lever (*b*) with respect to the shortest rear lever (*b* = 17).

Regarding the impact of the rear lever length (*b*), Figure 16a and Table 2 show the fatigue limit determined by the analytical model for b values of 17, 27, 37, and 47 mm and a front lever (*a*) value of 19 mm (identical to the clamps employed in the experimental tests, and the shortest possible for this application). In the graph, it can be observed that the number of cycles achieved for each clamp increases as the (b) value of rear lever increases, although this increase is not proportional to the increase in the value of the lever. As can be seen in Figure 16b, the fatigue limit increase obtained by enlarging the rear lever (with respect to the shortest rear lever *b* = 17 mm) is not proportional to the rear lever (*b*) relative increase with respect to the shortest rear lever. As the size of the rear lever *b* is increased, the fatigue resistance improvement is lower.

**Figure 17:**
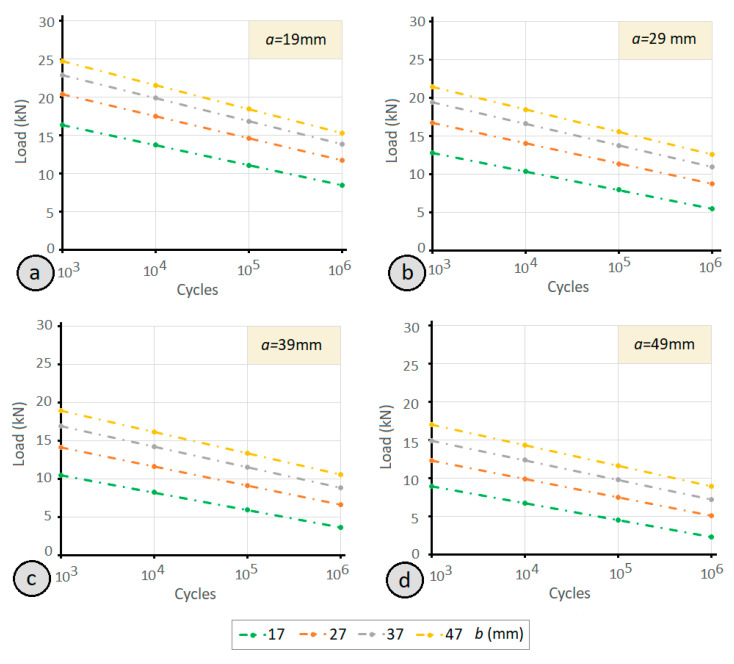
Variation of the fatigue limit of the clamp joint for different rear levers according to the size of the front lever and as a function of the number of life cycles: (**a**) front lever of 19 mm; (**b**) front lever of 29 mm; (**c**) front lever of 39 mm; (**d**) front lever of 49 mm.

As revealed by the analytical model results, when the value of front lever (*a*) increases, the fatigue limit decreases, while when the value of rear lever (*b*) increases, the fatigue limit increases. Moreover, the graphs in Figure 17 show the results change for different front levers (*a*), depending on the size of the rear lever (*b*). The combination that provides the greatest resistance to fatigue is the 19 mm front lever and 47 mm rear lever; that is, the case of the shortest front lever and the longest rear lever.

### 4.3. Comparison of Analytical Model Results and Laboratory Test Results

**Figure 18:**
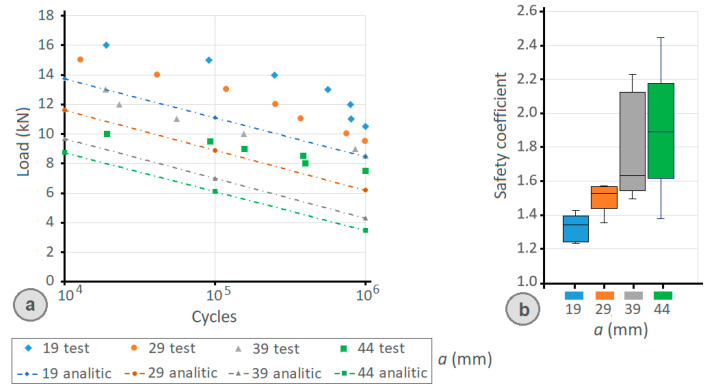
(**a**) Comparison between the results obtained with the analytical model and the results of the experimental tests; (**b**) box plot of the safety coefficient between the theoretical values according to the analytical model and the values according to the test.

Figure 18a shows the comparison between the analytical model results and the experimental results. As can be seen in the graphs, the maximum allowable fatigue load obtained with the analytical model is always lower than the value of the experimental tests, increasing the safety margin as the front lever increases.

Regarding the differences between the theoretical values according to the analytical model and the laboratory test, it is obtained an average factor of 1.63, with minimum values of 1.23. As shown in the box diagram of Figure 18b, for the 19 mm front lever clamp (19/17), the mean safety factor was 1.32, with a minimum value of 1.23. In the case of the 29 mm front lever clamp (29/17), the average safety factor was 1.51, with a minimum value of 1.36. For the 39 mm front lever clamp (39/17), the average safety factor was 1.77 with a minimum value of 1.49, and in the case of the clamp with a 44 mm front lever (44/17), the average safety factor was 1.90 with a minimum value of 1.38.

The data obtained in the laboratory show that the proposed analytical method is suitable for calculating the fatigue life of clamp joints, with results that guarantee to be on the safe side. In both cases (experimental results and analytical model results), it is verified that, as the size of the front lever (a) increases, the limit of resistance to fatigue of the joint decreases.

**Figure 19:**
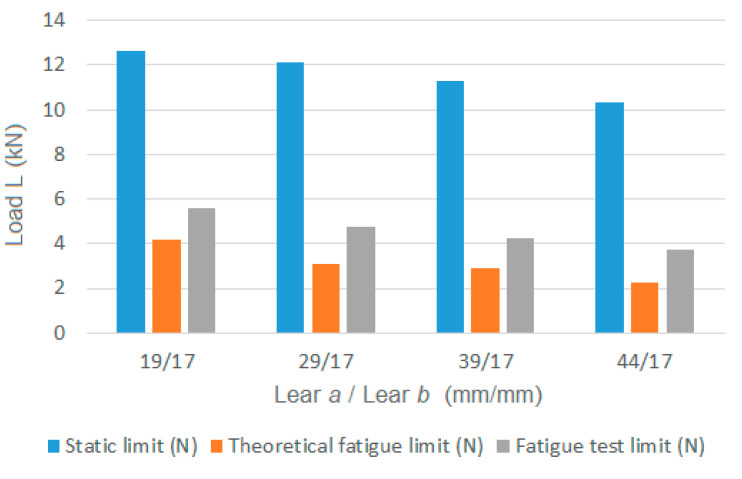
Graph of the maximum static load, theoretical fatigue limit and test fatigue limit for the different clamps tested.

For the geometric parameters of the clamp, the type of joint research, and according to the work of Cabaleiro et al. [2], the joint failure modes are type 2 and 3 (collapse of the joint due to the failure of the bolts). Based on these failure modes, the maximum static load value to be applied to the clamp (without exceeding the yield strength of the material) is shown in Figure 19. Table 3 and Figure 19 also show, in addition to the static strength, the theoretical fatigue limit according to the proposed analytical expression (for 10^6^ cycles) and the fatigue limit according to the experimental tests (for 10^6^ cycles).

As observed in Figure 19, the relationship between the maximum static load (without exceeding the bolt yield strength) and its theoretical fatigue resistance is 3.7 times lower on average, with a minimum observed value of 3.0. Regarding the relationship between the maximum static load (without exceeding the bolt yield strength) and its test fatigue resistance, this is 2.6 times lower on average, with a minimum observed value of 2.4. These results indicate that it is critical to consider the fatigue phenomenon in this type of joint when subjected to alternating loads for more than 1000 cycles.

## 5. Conclusions

Clamped joints represent a type of joint that allows the manufacture of fully removable and reconfigurable structures. This type of joint is also suitable when it is necessary to rehabilitate or modify a historical steel structure for new uses, reinforcing or modifying it with new profiles without the need to drill or weld the original structure. Nevertheless, it is necessary to consider that these structures can be subjected to dynamic stresses, eventually producing joint fatigue failures. There is a significant lack of studies on joint type, particularly on how they behave under fatigue loads as a function of the clamp levers’ geometric characteristics.

In this work, an analytical model has been proposed for the calculation of the number of cycles to failure and the fatigue limit of clamped joints as a function of the size of the clamp levers. The key conclusions of this work are as follows:

- The data obtained indicate that the joint strength can be reduced to one third when the joint is subjected to fatigue stresses, something that is essential to be considered in structures subjected to dynamic stresses, which can cause fatigue in the joints.
- In this work, an analytical model was proposed and used for a quick analysis of the number of cycles and the fatigue limit of clamped joints and their bolts, according to the size of the clamping levers. The experimental results prove that the maximum allowed fatigue load calculated with the analytical model is always lower than that achieved in the real tests.
- If the size of the front lever is increased, the fatigue resistance decreases, while for the rear lever, if its size is increased, the fatigue limit of the joint also increases. The combination that provides the greatest fatigue resistance of the clamp joint comprises of the shortest front lever and the longest rear lever.
- The experimental tests revealed that 96% of the time the bolt breakage occurred in the lower part, in the bolt–nut union area. The failure of one bolt at the head location was observed in the remaining 4%.

Finally, it can be concluded that this work contributes to an important advance in the clamp joint research, developing a straightforward analytical method of fatigue calculation that can be applied by engineers to calculate this type of joint.

## Figures and Tables

**Table 1:** Maximum load (*L*) to fatigue failure according to the size of the front clamp lever (*a*) and the number of cycles, for a fixed size of the rear clamp lever (b).

a/b (mm)	19/17	29/17	39/17	49/17
Cycles	Load L (N)	Load L (N)	Load L (N)	Load L (N)
1000	16,367	14,014	12,255	10,503
10,000	13,730	11,362	9590	7923
100,000	11,094	8710	6925	5344
1,000,000	8457	6057	4261	2764

**Table 2:** Maximum load (*L*) to fatigue failure according to the size of the rear clamp lever (*b*) and the number of cycles, for a fixed size of the front clamp lever (a).

a/b (mm)	19/17	19/27	19/37	19/47
Cycles	Load L (N)	Load L (N)	Load L (N)	Load L (N)
1000	16,367	20,344	22,900	24,682
10,000	13,729	17,468	19,872	21,547
100,000	11,092	14,593	16,844	18,413
1,000,000	8454	11,718	13,816	15,278

**Table 3:** Maximum static load, theoretical fatigue limit and test fatigue limit for the different clamps tested where (a) is the front clamp lever and (b) rear clamp lever.

a/b	Static Limit (N)	Theoretical Fatigue Limit (N)	Fatigue Test Limit (N)	Relationship (Static/Theoretical Fatigue)	Relationship (Static/Test Fatigue)
19/17	12,665	4229	5250	3.0	2.4
29/17	12,120	3192	4750	3.8	2.6
39/17	11,269	3240	4250	3.5	2.7
44/17	10,345	2277	3750	4.5	2.8

## Data Availability

The data presented in this study are available on request from the corresponding author.
